# Global, regional, and national burden of lip and oral cavity cancer attributable to smoking, 1990–2021: A secondary dataset analysis of GBD 2021

**DOI:** 10.18332/tid/219210

**Published:** 2026-05-22

**Authors:** Can Li, Jincheng Tang, Jingting Zhang, Tianhao Tong, Renyi Yang, Puhua Zeng

**Affiliations:** 1 Hunan Provincial Hospital of Integrated Traditional Chinese and Western Medicine, Hunan University of Chinese Medicine, Changsha, China; 2 Institute of Traditional Chinese Medicine Oncology, Hunan Academy of Chinese Medicine, Changsha, China

**Keywords:** lip and oral cavity cancer, smoking, disease burden, gender disparities, aging

## Abstract

**INTRODUCTION:**

Lip and oral cavity cancer (LOCC) poses a major public health challenge, primarily driven by smoking. This study evaluates the spatiotemporal trends of smoking-attributable LOCC using Global Burden of Disease (GBD) 2021 data, focusing on regional disparities and age-specific stagnations.

**METHODS:**

In this secondary dataset analysis of GBD 2021, incidence, mortality, and disability-adjusted life years of smoking-attributable LOCC across 204 countries and territories (1990–2021) were analyzed. Trends were quantified using estimated and average annual percentage changes (EAPC/AAPC) by region, gender, age, and sociodemographic index.

**RESULTS:**

In 2021, smoking accounted for 23.41% (95% UI: 20.1–26.5) of global LOCC deaths. Although the global age-standardized mortality rate (ASMR) decreased from 0.77 in 1990 to 0.56 per 100000 population in 2021 (EAPC= -1.09; 95% CI: -1.13 – -1.05), absolute deaths increased by 59.48% to 48773 (95% UI: 33622–63367). Notably, East Asia (EAPC=0.46; 95% CI: 0.19–0.64) and Central Sub-Saharan Africa (EAPC=0.12; 95% CI: 0.04–0.23) uniquely showed increasing ASMRs. Age-specific analysis revealed a plateaued improvement in the elderly, particularly the 75–79 years group (AAPC= -0.1; 95% CI: -0.5–0.3), contrasting with declines in younger groups. Males bore a higher burden, though female mortality declined faster (EAPC= -1.40 vs -1.10).

**CONCLUSIONS:**

While the global smoking-attributable LOCC burden is reducing, there are widening regional disparities and a statistical stagnation, specifically in the age group of 75–79 years. These divergent trends generate important hypotheses regarding the limits of uniform prevention. Persistent burdens imply context-specific factors, such as potential gene-environment interactions in East Asia and poly-tobacco use in South Asia, which warrant further investigation to achieve more equitable reductions in disease burden.

## INTRODUCTION

Lip and oral cavity cancers (LOCC), including cancers of the lip, tongue, and oral cavity, are the most common head and neck malignancies^1^. It is characterized by aggressive progression and poor prognosis, with a five-year survival rate of approximately 50%^2,3^. According to GLOBOCAN 2022, approximately 389846 new cases and 188438 deaths were reported globally in 2022; its incidence ranks 16th, and its mortality ranks 15th^1^. LOCC occupies an important position in the global cancer spectrum, posing a major threat to public health^4^. Although the comprehensive treatment methods, like surgery, radiotherapy, and chemotherapy, have made great progress^5^, patients with LOCC are often diagnosed in the late stage due to the delay of early screening technology and clinical detection; consequently, the efficacy of these treatment modalities remains limited for patients diagnosed at advanced stages^6^. Therefore, it is very important to prevent the occurrence of LOCC. Smoking is the most significant risk factor of LOCC^7^, and in some countries and regions, tobacco control has significantly reduced the occurrence of LOCC^8^, and non-smokers have a significantly lower risk of LOCC morbidity and mortality compared with smokers^9^. A systematic understanding of the epidemiological trends of LOCC caused by smoking can help policymakers develop targeted strategies. Although previous studies have analyzed the trend of incidence and mortality of LOCC caused by smoking and chewing tobacco and the correlation of the human development index from 2019 to 2020 based on GLOBOCAN^10^, there is still insufficient global data update on smoking-related LOCC.

Global Burden of Disease (GBD) Study 2021 included 204 countries between 1990 and 2021, with data on the incidence, mortality, and disability-adjusted life years (DALYs) of 371 diseases and injuries in 811 regions, as well as 88 related risk factors^11,12^. In 2021, the risk factors of LOCC included environmental, operational, and metabolic factors; 50.51% of age-standardized LOCC mortality was attributable to all quantified risk factors in GBD 2021, with smoking contributing 23.41%, almost equal to the combined contribution of other risk factors^12^. This study analyzed smoking-related LOCC burden by sex, age, region, country, and sociodemographic index (SDI) based on GBD 2021 data.

## METHODS

### Data resources

This study is a secondary dataset analysis of the Global Burden of Disease (GBD) 2021 study. The timeframe of the analysis spanned 1990 to 2021. The eligibility criteria for data inclusion were comprehensive global data encompassing 204 countries and territories, specifically extracting the incidence, mortality, and disability-adjusted life years (DALYs) of LOCC directly attributable to smoking.

The detailed methods of the GBD 2021 estimation process have been previously described in the literature^11-13^. ‘Death’, ‘DALY’, age-standardized mortality rate (ASMR), and age-standardized disability-adjusted life years rate (ASDR) were used as the measurement index, ‘LOCC’ as the cause, and ‘smoking’ as a risk factor. The study explored the global burden of LOCC in 21 regions, 5 SDI regions, and 204 countries worldwide and utilized de-identified publicly available data, following the ‘Guidelines for Accurate and Transparent Health Estimation Reports’^14^.

### Definitions

GBD 2021 research uses the International Classification of Diseases (ICD) codes to define diseases, specifically ICD-915^15^ and ICD-1016^16^. In this study, LOCC is classified under ICD-9 code 140-145 and ICD-10 code C00-C06. Smoking is classified as a behavioral risk, as a subcategory of tobacco, defined as the current or past active use of any tobacco product (excluding chewing tobacco and passive secondhand smoke). Exposure is measured by the number of cigarettes smoked daily and the cumulative years of smoking^12,17^.

‘Death’ refers to the number of deaths related to diseases within a specific period. ‘DALYs’ is the sum of years of life lost (YLLs) and years of life lost due to disability (YLDs). The specific formula is: YLL=number of deaths × standard life expectancy at the age of death, YLD=prevalence rate × disability weight^11^. Death and DALYs are both key indicators for assessing the burden of disease. This analysis presents all estimated values in the form of counts, age-specific rates, and age-standardized rates per 100000 population (ASR), to measure the burden of disease. GBD 2021 divides the world into 21 regions based on geographical differences, including Central Asia, Central Sahara Africa, Central Latin America, Oceania, and Western Europe, and includes data from 204 countries and regions. Age groups are divided into 20 groups, ranging from <5 years to ≥95 years, with each group representing a 5-year increase. The sociodemographic index (SDI) quantifies the level of economic and social development of countries and regions, which is closely related to health outcomes, with the SDI ranging from 0 to 1, where lower values indicate poorer urban development and higher values indicate greater socio-economic progress. Based on lagged income per capita, total fertility rate for ages <25 years, and average years of education for people aged ≥15 years, 204 countries and regions were classified into five SDI categories: high (0.805129–1), high-middle (0.689504–0.805129), middle (0.607679–0.689504), low-middle (0.454743–0.607679), and low (0–0.454743).

The GBD Cause of Death Ensemble modeling (CODEm) was used to generate mortality estimates for different genders, ages, time periods, and regions^18^. Based on GBD 2021 global population data, age-standardized mortality rate (ASMR) and age-standardized disability-adjusted life year rate (ASDR) were used to quantify smoking-related LOCC burden in different regions to eliminate biases caused by differences in population and age composition. For deaths, DALYs, ASMR, and ASDR, 95% uncertainty intervals (UI) were derived by drawing the 25th and 975th values out of 1000 from the uncertainty distribution.

The estimated annual percentage change (EAPC) and its corresponding 95% confidence interval (CI) were calculated to describe the trends of smoking-related LOCC age-standardized rate (ASR) from 1990 to 2021. The trends were considered statistically significant if the 95% CI of the EAPC did not contain zero; specifically, an ASR was deemed to be increasing if the lower boundary of the 95% CI was >0, and decreasing if the upper boundary was <0. Furthermore, to analyze trends across different time periods, such as the entire study period versus the most recent five years, the average annual percentage change (AAPC) was calculated. This allows for a more detailed comparison of long-term and recent secular trends in the data. All analyses and visualizations were performed using the software R (version 4.2.2)^19^ and R Studio, and a two-tailed p<0.05 was considered statistically significant. Furthermore, a temporal trend was defined as a ‘stagnation’ if the 95% CI of the EAPC or AAPC included zero, indicating no statistically significant increase or decrease over the specified period.

## RESULTS

### Global deaths and DALYs

In 2021, the number of smoking-related LOCC deaths worldwide was 48772.68, an increase of 59.48% compared to 1990. The ASMR from 1990 to 2021 decreased from 0.77/100000 to 0.56/100000. Additionally, the EAPC of ASMR from 1990 to 2021 was -1.091, with a total decrease of 27.27%.

In 2021, the number of LOCC deaths caused by smoking among females was 4750.66, and among males was 44022.02. Males accounted for 35.12% of deaths, and among females it was 6.65%. The ASMR for males was 1.08/100000, and for females was 0.16/100000, indicating a significant gender difference. From 1990 to 2021, the EAPC of ASMR for females was -1.400, and for males was -1.104, with a faster decline rate than for females. Despite the increase in LOCC deaths caused by smoking, the ASMR showed a decreasing trend, with the number of deaths, death percentages, and ASMR among females all significantly lower than among males.

Globally, the number of DALYs increased by 47.89%, from 887463.80 in 1990 to 1312511.38. The ASDR decreased from 21.29 per 100000 to 14.91 per 100000, with a percent decline of 29.97%, and the EAPC was -1.241 in 2021. The number of DALYs and ASDR in males were 1200789.93 and 28.43/100000, respectively, which were significantly higher than those in females (111721.45 and 2.43/100000). The EAPC of ASDR in males was -1.221, and that in females was -1.526. The percentage increase of DALYs in males (49.17%) was much higher than that in females (35.45%) (Table 1).

**Table 1:** Global deaths and disability-adjusted life years (DALYs) of smoking-attributable LOCC (1990–2021)

	*All*	*Female*	*Male*
**1990**			
Deaths (95% UI) DALYs (95% UI) ASMR/100000 persons (95% UI) ASDR/100000 persons (95% UI)	305811 (686229.20–791982.05) 887463.80 (641442.69–1120445.23) 0.77 (0.55–0.98) 21.29 (15.35–26.91)	3232.5 (2136.11–4452.14) 82480.85 (55135.30–112223.66) 0.16 (0.10–0.22) 3.86 (2.58–5.27)	27349.43 (19810.44–34452.63) 804982.95 (585388.85–1012195.26) 1.48 (1.07–1.88) 40.33 (29.26–50.88)
**2021**			
Deaths (95% UI) DALYs (95% UI) ASMR/100000 persons (95% UI) ASDR/100000 persons (95% UI)	48772.68 (33622.36–63367.06) 1312511.38 (910638.11–1697046.15) 0.56 (0.39–0.73) 14.91 (10.34–19.27)	4750.66 (3008.93–6367.06) 111721.45 (72361.80–157418.06) 0.10 (0.06–0.15) 2.43 (3.42–1.57)	44022.02 (30576.20–56922.84) 1200789.93 (836281.69–1558488.68) 1.08 (0.75–1.40) 28.43 (19.79–36.93)
**1990–2021**			
PC of deaths (95% CI) PC of DALYs (95% CI) EAPC of ASMR (95% CI) EAPC of ASDR (95% CI)	-26.856 (-36.664–19.042) -29.954 (-39.706–22.299) -1.091 (-1.132–1.050) -1.241 (-1.280–1.201)	-34.678 (-40.579–27.370) -37.176 (-42.727–30.604) -1.400 (-1.485–1.315) -1.526 (-1.604–1.447)	-27.064 (-37.882–18.668) -29.501 (-40.152–21.155) -1.104 (-1.146–1.061) -1.221 (-1.259–1.182)

LOCC: lip and oral cavity cancer. ASMR: age-standardized mortality rate. ASDR: age-standardized disability-adjusted life-year rate. PC: percentage change. EAPC: estimated annual percentage change. UI: uncertainty interval. CI: confidence interval. Study design: A secondary dataset analysis. Setting and sample size: Global populations across 204 countries and territories. Data source: The Global Burden of Disease (GBD) 2021 study.

**Figure 1:**
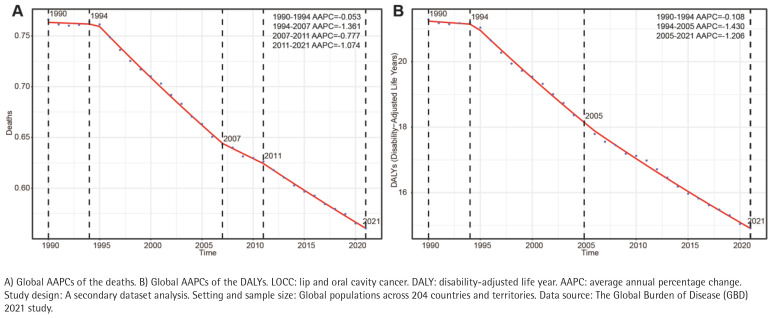
Global AAPCs of deaths and DALYs for smoking-attributable LOCC (1990–2021)

Between 1990 and 1994, mortality rates remained relatively stable, with a very limited decline; from 1994 to 2007, there was a rapid decline; from 2007 to 2011, the decline rate slowed down; and from 2011 to 2021, the decline rate accelerated again (Figure 1A). The DALY rate showed a limited decline between 1990 and 1994; the fastest decline was between 1994 and 2005; and from 2005 to 2021, it maintained a relatively stable downward trend (Figure 1 B).

### Regional deaths and DALYs

**Figure 2:**
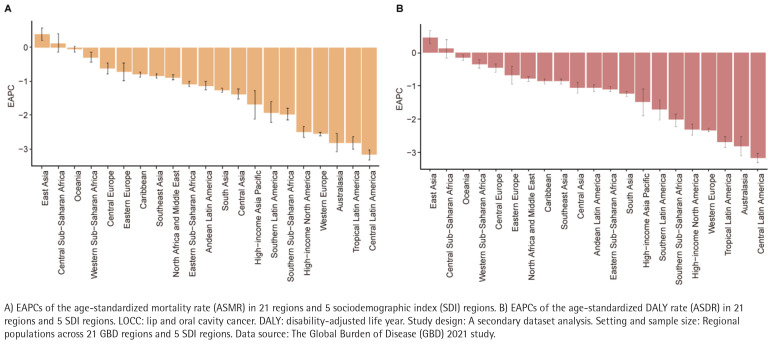
Estimated annual percentage changes (EAPCs) of age-standardized rates (ASRs) for smoking-attributable LOCC (1990–2021)

At the regional level, in 2021, the top three regions with the highest number of smoking-related LOCC deaths were South Asia (16894.05), East Asia and the Pacific (16344.57), and Southeast Asia (15726.17). The region with the highest ASMR was South Asia (1.14/100000). From 1990 to 2021, only two regions, East Asia (EAPC= 0.46) and Sub-Saharan Africa central (EAPC=0.12), experienced positive EAPC, indicating that the overall mortality rate of smoking-related LOCC is on the rise, with the highest increase in East Asia. In contrast, the EAPC of ASMR in the other 19 GBD regions was negative, reflecting a general downward trend. The largest decline in ASMR was in middle Latin America (EAPC= -3.163), followed by the entire Latin America and Oceania, with their EAPCs both less than -2.5 (Figure 2A). In addition, in 2021, the ASMR of smoking-related LOCC in all regions was significantly higher in males than in females, with the highest ASMR in males in Eastern Europe and in females in Central Europe (Supplementary file Figure S3A).

Similarly, in 2021, the highest DALY caused by smoking-related LOCC is in South Asia (459465.21), with the highest ASDR (31.031/100000) in Eastern Europe, followed by Central Europe (30.639/100000) and South Asia (29.190/100000), far higher than other regions. From 1990 to 2021, the ASDR in East Asia increased the most (EAPC=0.394), followed by Sub-Saharan Africa central (EAPC=0.134), with only these two regions experiencing positive growth (Figure 2B). The largest decline was in middle Latin America (EAPC= -3.168). Notably, in 2021, the ASDR of smoking-related LOCCs in all regions was significantly higher in males than in females, with the highest ASDR in females in high-income Central Europe (6.573/100000) and in males in Central Europe (68.388/100000) (Supplementary file Figure S3B).

### National deaths and DALYs

At the country level, in 2021, the country with the highest number of smoking-related LOCC deaths was China (10144.44). The death toll in China significantly exceeded that of other nations, contrasting sharply with the 1000 to 3000 deaths reported in the next highest-ranking countries. The countries with the highest ASMR were Pakistan (2.775/100000), Seychelles (2.309/100000), and Taiwan (2.186/100000) (Supplementary file Figure S1A). From 1990 to 2021, the ASMR of smoking-related LOCC in 48 countries showed an overall upward trend, while in 156 countries it showed a downward trend. The country with the fastest ASMR increase was Cape Verde (EAPC=6.672), followed by the Northern Mariana Islands (EAPC=2.788) and Guam (EAPC=2.657). Conversely, the countries with the fastest ASMR decrease were Colombia (EAPC= -4.316), Norway (EAPC= -3.614), Mexico (EAPC= -3.612), France (EAPC= -3.554), and Ireland (EAPC= -3.483) (Supplementary file Figure S4A).

In 2021, the countries with the highest number of smoking-related LOCC cancer DALYs were India (311055.48) and China (259703.27). Similar to ASMR, the country with the highest ASDR was Pakistan (71.18/100000), followed by Taiwan (69.04/100000) (Supplementary file Figure S1B). Between 1990 and 2021, the ASDR increased in 49 countries and decreased in 155 countries. The largest ASDR increase was seen in Cabo Verde (EAPC=6.738), followed by Guam (EAPC=2.916), Northern Mariana Islands (EAPC=2.794), and Guinea-Bissau (EAPC=2.437). In contrast, Colombia (EAPC= -4.442) experienced the largest decrease in ASDR, followed by France (EAPC= -3.675) and Norway (EAPC= -3.660) (Supplementary file Figure S4B).

### Based on SDI, the burden of LOCC

At the SDI regional level, in 2021, the middle SDI region recorded the highest number of smoking-related LOCC deaths (14036.85) and DALYs (373676.15), followed by the middle-low and middle-high regions. Similarly, the middle-low SDI region also had the highest ASMR (0.93/100000) and ASDR (23.61/100000). Moreover, from 1990 to 2021, the ASMR and ASDR of smoking-related LOCC in all five SDI regions showed varying degrees of decline, with the high SDI region experiencing the most significant decrease. Specifically, the EAPCs for ASMR and ASDR in this region were -1.697 and -1.849, respectively. In 2021, the high SDI region and the middle-low SDI region had the highest ASMR and ASDR for females, with ASMR of 0.15/100000 and 0.16/100000, and ASDR of 3.95/100000 and 3.72/100000, respectively. In the middle-low SDI region, the ASMR and ASDR of males were also higher than those in other SDI regions. Additionally, from 1990 to 2021, the decline in ASMR and ASDR for males in the high-middle and high SDI regions was significantly greater than that for females (Supplementary file Figures S2 and S5).

### Smoking-related LOCC burden by age and sex

In 2021, the age-specific burden of smoking-related LOCC exhibited distinct patterns between genders. Regarding mortality, the trends for absolute numbers and age-specific rates differed. The absolute number of deaths rose steadily with age, reaching a single peak in the age group of 65–69 years for both males (7421.31) and females (685.77), before declining in the oldest age groups. Conversely, age-specific mortality rates continued to increase progressively with advancing age, reaching their highest levels in the oldest populations.

**Figure 3:**
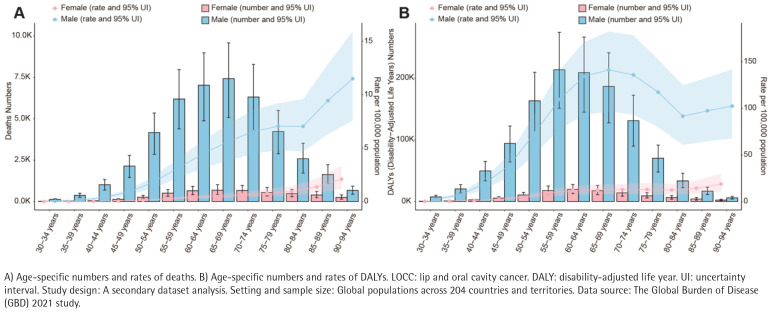
Age-specific numbers and rates of deaths and DALYs for smoking-attributable LOCC by gender (2021)

In contrast, trends in age-specific DALY rates diverged significantly by sex. Females displayed a continuous upward trend, with rates increasing progressively to a peak in the age group 90–94 years (18.72 per 100000). However, males exhibited a complex fluctuating pattern: the age-specific DALY rate for males peaked initially in the age group of 65–69 years at 141.25 per 100000, declined through the age groups of 75–89 years, and notably increased again to 102.38 per 100000 in the age group 90–94 years. Across all age groups, a profound gender disparity was evident, with males consistently bearing a significantly higher burden of both deaths and DALYs compared to females. Additionally, the disease burden remained negligible in individuals under 30 years of age (Figure 3).

For the three decades from 1990 to 2021, the AAPC for both death and DALY rates were negative for all age groups, and none of the 95% confidence intervals (CIs) contained zero, indicating that the downward trend was statistically significant. The largest decline was seen in the midlife group, with the most pronounced decline in the midlife group between 35 and 54 years of age over this period, with AAPC generally ranging from -1.6% to -1.8%. From the age of 60 years, the rate of decline gradually slows with increasing age; for example, the AAPC for mortality in the 90–94 years group is -0.4%, which is significantly lower than in the middle-aged group. In the secular trend, the AAPC for mortality and the AAPC for DALYs rates were very close across age groups (Table 2).

**Table 2:** Average annual percentage changes (AAPCs) of mortality and DALYs rates for smoking-attributable LOCC by age group (1990–2021)

*Age (years)*	*AAPC of deaths rate* *% (95% CI)*		*AAPC of DALYs rate* *% (95% CI)*	
	*1990–2021*	*2017–2021*	*1990–2021*	*2017–2021*
30–34	-1.6 (-1.7 – -1.4)	-1.1 (-1.8 – -0.4)	-1.5 (-1.7 – -1.4)	-1.1 (-1.8 – -0.4)
35–39	-1.7 (-1.8 – -1.5)	-2.5 (-3.8 – -1.1)	-1.7 (-1.8 – -1.5)	-2.4 (-3.8 – -1.1)
40–44	-1.7 (-1.8 – -1.6)	-0.9 (-1.4 – -0.5)	-1.7 (-1.8 – -1.6)	-0.9 (-1.4 – -0.5)
45–49	-1.8 (-1.9 – -1.7)	-1.2 (-1.9 – -0.6)	-1.8 (-1.9 – -1.7)	-1.2 (-1.9 – -0.6)
50–54	-1.6 (-1.7 – -1.5)	-0.8 (-1.1 – -0.5)	-1.7 (-1.8 – -1.6)	-0.9 (-0.9 – -0.8)
55–59	-1.2 (-1.3 – -1.1)	-2.7 (-3.7 – -1.6)	-1.3 (-1.4 – -1.2)	-2.6 (-3.7 – -1.6)
60–64	-1.1 (-1.2 – -0.9)	-0.9 (-1.1 – -0.7)	-1.1 (-1.2 – -1.0)	-0.9 (-1.0 – -0.7)
65–69	-0.9 (-1.0 – -0.7)	-1.0 (-1.4 – -0.6)	-0.9 (-1.0 – -0.8)	-0.4 (-1.4–0.5)
70–74	-0.7 (-0.8 – -0.6)	-0.5 (-0.7 – -0.4)	-0.7 (-0.7 – -0.6)	-1.0 (-1.4 – -0.6)
75–79	-0.6 (-0.6 – -0.5)	-0.1 (-0.5–0.3)	-0.6 (-0.6 – -0.5)	-0.1 (-0.4–0.3)
80–84	-0.5 (-0.6 – -0.4)	-1.0 (-1.9 – -0.2)	-0.5 (-0.6 – -0.4)	-0.9 (-1.9–0.2)
85–89	-0.4 (-0.5 – -0.2)	-1.1 (-2.7–0.6)	-0.3 (-0.5 – -0.2)	-0.8 (-2.8–1.2)
90–94	-0.4 (-0.5 – -0.2)	-0.8 (-2.2–0.6)	-0.3 (-0.6 – -0.1)	-0.8 (-2.2–0.6)

LOCC: lip and oral cavity cancer. DALY: disability-adjusted life year. CI: confidence interval. Study design: A secondary dataset analysis. Setting and sample size: Global populations across 204 countries and territories. Data source: The Global Burden of Disease (GBD) 2021 study.

In the last five years, it is worth noting that the two age groups of 35–39 and 55–59 years have very significant downward trends in the recent period, even exceeding the long-term average. For example, the AAPC for the age group of 55–59 years reached -2.7%. The rate of decline in many age groups (e.g. 30–34, 40–44, 50–54 years) has slowed in the last five years compared to long-term trends. The plateaued improvement in the oldest age groups, is an important warning sign. In those aged >75 years, the downward trend almost disappeared (Table 2).

## DISCUSSION

Using the latest GBD 2021 data, this study provides a comprehensive analysis of the epidemiological trends of smoking-attributable LOCC at global, regional, and national levels. In 2021, smoking remained the leading risk factor, accounting for 23.41% of LOCC deaths, followed by alcohol use (19.21%) and chewing tobacco (14.28%). From 1990 to 2021, the global absolute number of deaths and DALYs increased significantly, a trend largely driven by global population growth and accelerated population aging^20^.

While the global age-standardized mortality rate (ASMR) has generally declined, the present age-specific analysis reveals a critical warning sign: improvement has plateaued in the oldest age groups (>75 years). Unlike younger age groups, the elderly have shown a negligible decline in mortality rates over the past three decades. This plateauing trend is likely attributable to the cumulative nature of DNA damage from long-term smoking, which becomes less reversible with age^21^. Furthermore, many patients do not undergo health screening until the middle or advanced age, when the disease is often advanced and malignant development is faster^22^. Additionally, the exposure to over 70 carcinogens in tobacco induces chronic inflammation, oxidative stress, and immune suppression, altering the tumor microenvironment and compounding risks in the elderly^23^.

Despite these challenges, the overall downward trend in ASMR and ASDR is encouraging and likely reflects advances in medical technology and stricter tobacco control policies. Recent studies indicate that smoking cessation significantly reduces the risk of postoperative recurrence and malignant transformation^24^, while improving survival rates and treatment responses in patients undergoing chemoradiotherapy^25^. Personalized cessation interventions have also been shown to improve physiological outcomes in oral cancer patients^26^. The International Agency for Research on Cancer (IARC) confirms that targeted screening among high-risk groups by clinical oral examination has shown mortality benefits in some settings. This is supported by a meta-analysis indicating that screening high-risk groups could reduce oral cancer mortality by 26% and advanced cases by 19%^27^. For instance, Taiwan introduced a biennial oral mucosal screening program for high-risk individuals in 1999, which has been significantly associated with reduced mortality^28^. Similarly, the Trivandrum trial in India demonstrated a significant 24% reduction in mortality among high-risk groups (smokers/drinkers), despite non-significant results in the general population. However, current evidence remains insufficient to support universal screening for asymptomatic general populations^29^, as the potential harms from false positives may outweigh the benefits in low-incidence settings.

Significant regional disparities persist. Notably, East Asia and Central Sub-Saharan Africa were the only regions to exhibit an increase in ASMR and ASDR from 1990 to 2021, diverging from the decline seen in 19 other regions. This phenomenon can be interpreted through the ‘four-stage model’ of the tobacco epidemic^30^. While North America and Western Europe have reached the fourth stage (declining prevalence and mortality), East Asia appears to be transitioning between the second and third stages, characterized by high male smoking prevalence and a lagged rise in mortality due to the long incubation period of cancer^31^.

Crucially, this rising burden in East Asia is likely exacerbated by a unique biological interaction. A high prevalence of the ALDH2 gene mutation exists in East Asian populations^32^, which impairs acetaldehyde metabolism. When combined with the widespread co-behavior of smoking and alcohol consumption in this region, the synergistic carcinogenic effect is significantly amplified^33^. This gene-environment interaction may explain why East Asia’s mortality trend defies the global decline. Therefore, countries that implement comprehensive and stringent tobacco control policies at an earlier stage, accompanied by widespread health education and socio-economic transformation, tend to transition more rapidly from the high-burden second and third stages of the tobacco epidemic to the declining fourth stage^32^.

South Asia recorded the highest absolute mortality burden. It is critical to interpret this finding within the region’s complex landscape of tobacco use. While the present analysis isolates the burden of smoking (excluding chewing tobacco), poly-tobacco use is pervasive in South Asia. Many individuals are dual users who smoke cigarettes/bidis and consume smokeless tobacco (e.g. betel quid). This dual usage likely creates a multiplicative carcinogenic risk, suggesting that the high mortality attributed to smoking in this region is partially driven by the compounded toxicity of multiple tobacco products^34^.

The observed disparities, particularly the plateauing trend in the elderly and the rising burden in East Asia, provide important hypothesis-generating implications rather than evidence-based clinical recommendations. For instance, the plateauing trend in older populations generates the hypothesis that late-life screening approaches for long-term smokers may need re-evaluation. Furthermore, the regional outliers imply that underlying gene-environment interactions, such as ALDH2 deficiency combined with alcohol use in East Asia, and specific cultural behaviors, like poly-tobacco use in South Asia, may modify the risk landscape. Future individual-level clinical and epidemiological studies are required to confirm causality and design appropriate interventions.

### Limitations

This study has several limitations. First, GBD estimates rely heavily on the quality of raw data submitted by countries. The quality and coverage of these data are unevenly distributed globally; while high-income countries often have well-established population-based cancer registries, many low- and middle-income countries have weak registries with sparse, incomplete, or biased data. Second, the diagnostic classification is homogeneous. The GBD database analyzes ‘lip and oral cavity cancer’ as a single disease entity; however, clinically and pathologically, this is a heterogeneous group covering tumors in different anatomical subsites (e.g. lip, tongue, buccal mucosa) with distinct epidemiological characteristics and prognoses. Third, changes in smoking exposure have a delayed effect on LOCC incidence, creating a lag in the observable impact of recent tobacco control interventions. Fourth, while regional disparities were identified, risk assessment frameworks may struggle to adequately quantify the complex biological synergies among concurrent risk factors, such as smoking combined with alcohol or betel quid chewing, potentially underestimating the burden in populations with multiple high-risk behaviors. Fifth, smoking-related data are often derived from subjective patient reports, which can introduce self-reporting bias. Finally, a key limitation that must be acknowledged is that this study relies on secondary, observational data from the GBD database. As an ecological analysis, these findings are descriptive and do not permit causal inference regarding the relationships between smoking, genetic or regional factors, and LOCC outcomes at the individual level.

## CONCLUSIONS

This secondary dataset analysis of GBD 2021 demonstrates that despite overall global declines in age-standardized mortality and DALY rates, the reduction in the burden of smoking-attributable LOCC is characterized by pronounced regional and demographic inequalities. Reductions in disease burden decelerate with advancing age, approaching a statistical stagnation among individuals aged 75–79 years. Concurrently, East Asia and Central Sub-Saharan Africa continue to diverge from the global downward trend, exhibiting sustained increases. These findings highlight the uneven benefits of past tobacco control efforts and cautiously underscore the need to explore age-specific and context-specific dimensions in future research to achieve more equitable reductions in the smoking-related disease burden.

## Supplementary Material



## Data Availability

The data supporting this research are available from the following source: The Global Health Data Exchange (GHDx) query tool (https://ghdx.healthdata.org/gbd-2021).
