# Supplementary material for: Global, regional, and national burden of lip and oral cavity cancer attributable to smoking, 1990–2021: A secondary dataset analysis of GBD 2021
Source: Tob Induc Dis. 2026 May 22;24:10.18332/tid/219210. doi: 10.18332/tid/219210 (PMC13200591; doi:10.18332/tid/219210)
Supplement: Supplementary file 1 [file TID-24-67-s1.pdf]

Supplementary Material

Supplementary Figure S1. Correlation between SDI and ASRs for smoking-attributable LOCC at the national level (2021)

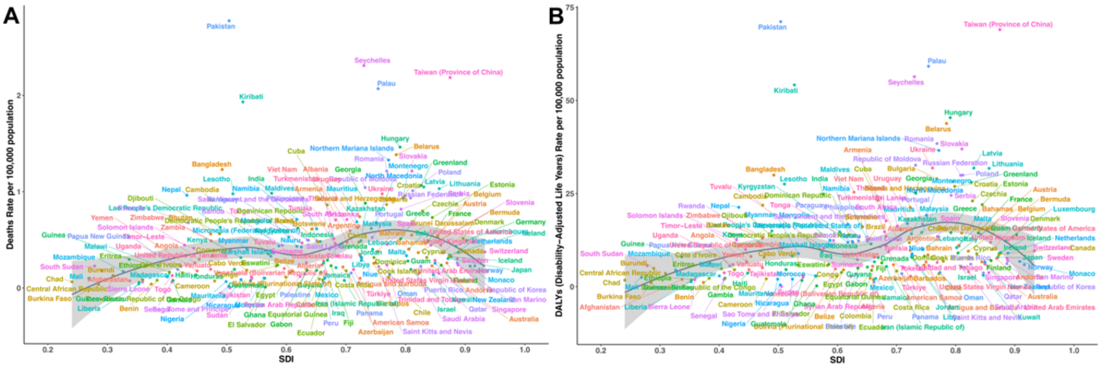

Note: (A) Correlation between the age-standardized mortality rate (ASMR) and SDI across 204 countries and territories. (B) Correlation between the age-standardized DALY rate (ASDR) and SDI across 204 countries and territories. LOCC, lip and oral cavity cancer; SDI, socio-demographic index; ASR, age-standardized rate; DALY, disability-adjusted life year. Study design: A secondary dataset analysis. Setting and sample size: National populations across 204 countries and territories. Data source: The Global Burden of Disease (GBD) 2021 study.

Supplementary Figure S2. EAPCs of ASRs for smoking-attributable LOCC across 5 SDI regions (1990–2021).

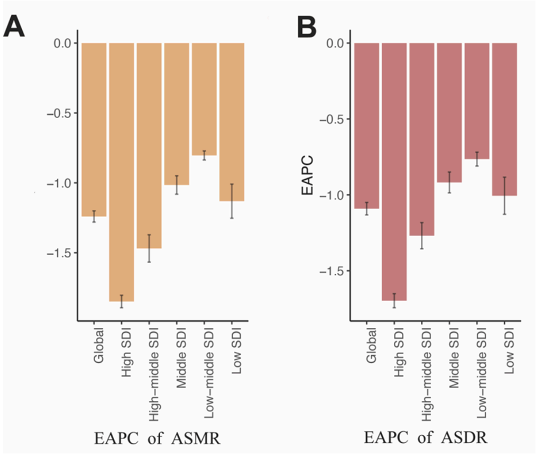

Note: (A) EAPCs of the age-standardized mortality rate (ASMR). (B) EAPCs of the age-standardized DALY rate (ASDR). LOCC, lip and oral cavity cancer; EAPC, estimated annual percentage change; ASR, age-standardized rate; SDI, socio-demographic index; DALY, disability-adjusted life year. Study design: A secondary dataset analysis. Setting and sample size: Regional populations across 5 SDI regions. Data source: The Global Burden of Disease (GBD) 2021 study.

Supplementary Figure S3. Gender differences in ASRs for smoking-attributable LOCC (2021).

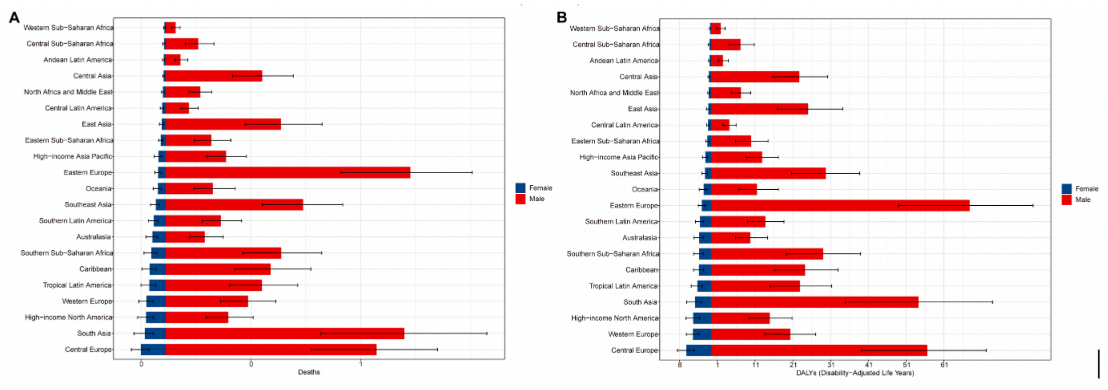

Note: (A) Gender differences in age-standardized mortality rate (ASMR) across 21 regions. (B) Gender differences in age-standardized DALY rate (ASDR) across 21 regions. LOCC, lip and oral cavity cancer; ASR, age-standardized rate; DALY, disability-adjusted life year. Study design: A secondary dataset analysis. Setting and sample size: Regional populations across 21 GBD regions. Data source: The Global Burden of Disease (GBD) 2021 study.

Supplementary Figure S4. EAPCs of ASRs for smoking-attributable LOCC at the national level (1990–2021)

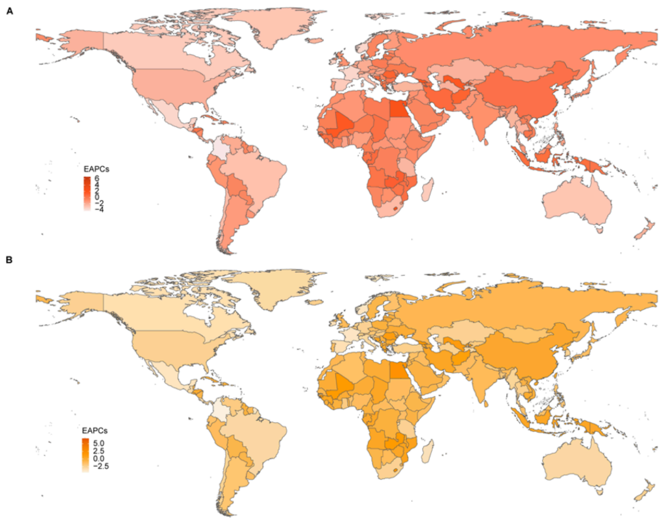

Note: (A) EAPCs of the age-standardized mortality rate (ASMR) in 204 countries and territories. (B) EAPCs of the age-standardized DALY rate (ASDR) in 204 countries and territories. LOCC, lip and oral cavity cancer; ASR, age-standardized rate; DALY, disability-adjusted life year; EAPC, estimated annual percentage change. Study design: A secondary dataset analysis. Setting and sample size: National populations across 204 countries and territories. Data source: The Global Burden of Disease (GBD) 2021 study.

Supplementary Figure S5. ASRs for smoking-attributable LOCC across 21 regions by SDI

(1990–2021)

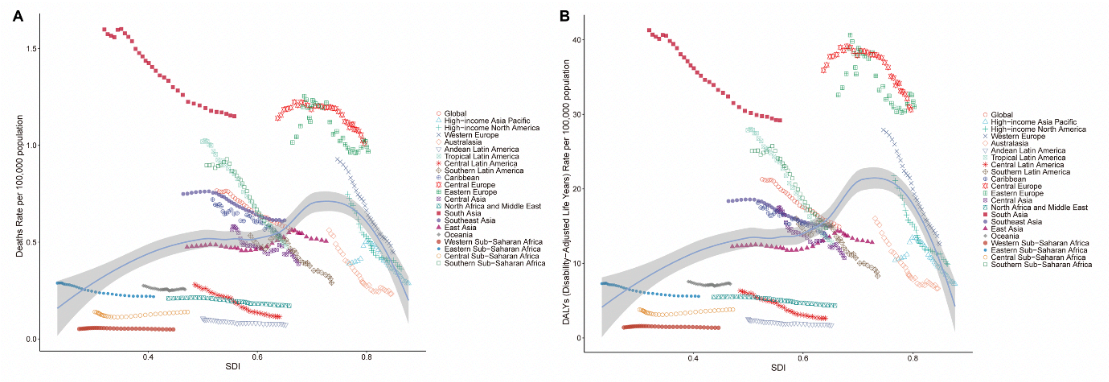

Note: (A) Age-standardized mortality rates (ASMRs) according to the SDI. (B) Age-standardized DALY rates (ASDRs) according to the SDI. LOCC, lip and oral cavity cancer; ASR, age-standardized rate; DALY, disability-adjusted life year; SDI, socio-demographic index. Study design: A secondary dataset analysis. Setting and sample size: Regional populations across 21 GBD regions. Data source: The Global Burden of Disease (GBD) 2021 study.

© 2026 Li C. et al.
